# 固相萃取-液相色谱-串联质谱法测定海水中软骨藻酸

**DOI:** 10.3724/SP.J.1123.2021.02026

**Published:** 2021-08-08

**Authors:** Jiuming WANG, Junhui CHEN, Jianbo YANG, Xiuping HE, Yuning WANG, Baodong WANG

**Affiliations:** 1.自然资源部第一海洋研究所, 自然资源部海洋生态环境科学与技术重点实验室, 海洋生物资源与环境研究中心, 山东 青岛 266061; 1. Marine Bioresource and Environment Research Center, Key Laboratory of Marine Eco-Environmental Science and Technology, the First Institute of Oceanography, Ministry of Natural Resources, Qingdao 266061, China; 2.青岛海洋科学与技术试点国家实验室, 海洋生态与环境科学功能实验室, 山东 青岛 266071; 2. Laboratory for Marine Ecology and Environmental Science, Pilot National Laboratory for Marine Science and Technology (Qingdao), Qingdao 266071, China

**Keywords:** 液相色谱-串联质谱, 固相萃取, 软骨藻酸, 海水, liquid chromatography-tandem mass spectrometry (LC-MS/MS), solid phase extraction (SPE), domoic acid (DA), seawater

## Abstract

软骨藻酸(domoic acid, DA)是一种由海洋硅藻产生的生物毒素,具有强烈的神经毒性,近海水环境中的DA严重威胁海洋渔业生物和人类健康,因此对近海水环境中的DA进行有效监测至关重要。该文基于固相萃取-液相色谱-串联质谱联用技术(SPE-LC-MS/MS),建立了适用于海水中痕量、超痕量DA的检测方法。针对近海水生环境中DA浓度相对较高的情况下,采用在线SPE-LC-MS/MS检测模式,可减少前处理过程,提高样品的分析效率。离线SPE结合在线SPE-LC-MS/MS可实现大洋和极地海水中含量更低的DA的检测。通过对在线固相萃取条件和液相色谱、质谱条件的优化,海水样品经过滤和酸化简单处理后直接进样0.6 mL进行在线SPE-LC-MS/MS检测,DA在10.0~500.0 ng/L范围内线性关系良好(线性相关系数*R*^2^=0.9992),检出限(LOD)和定量限(LOQ)分别为4.0和10.0 ng/L,并且具有较好的方法回收率(≥81.0%)和精密度(RSD≤4.2%),表明方法可用于近海海水中痕量DA的检测。通过对离线固相萃取柱的选择和酸化条件的优化,80.0 mL海水样品经离线HLB固相萃取柱富集后,进行在线SPE-LC-MS/MS检测,DA在0.3~50.0 ng/L范围内线性关系良好(*R*^2^=0.9990),回收率(≥69.2%)和精密度(RSD≤4.4%)较好,LOD和LOQ分别为0.1和0.3 ng/L,说明方法的灵敏度较直接进样法大幅提升,实现了海水中超痕量DA的准确测定。这两种检测方法操作简单,样品用量小,灵敏度高,可满足近海养殖区及远岸海水中DA监测的要求。

## Abstract (en)

Domoic acid (DA) can poison or even be fatal to marine mammals, and poses a potential risk to human health via transmission through the food chain. The level of DA in seawater will affect the safety of seafood. Therefore, a powerful method for the detection of DA in seawater, especially in the coastal mariculture zone, is needed. In order to identify different concentration levels of DA in real seawater, in this study, a method was established for the determination of trace DA in seawater by SPE-LC-MS/MS. First, the LC-MS/MS instrument and sample pretreatment conditions were optimized. Subsequently, DA was separated on a 5 TC-C18 (2) analytical column (150 mm×4.6 mm, 5 μm), and multiple reaction monitoring (MRM) was conducted in the positive electrospray ionization mode. For off-line SPE, the HLB cartridge could enrich DA in seawater. The best enrichment of DA was obtained after adding 0.32 mL formic acid to an 80.0 mL seawater sample. Four on-line SPE columns from Agilent, namely, 5 TC-C18(2) (12.5 mm×4.6 mm, 5 μm), Zorbax Eclipse Plus-C18 (12.5 mm×2.1 mm, 5 μm), Zorbax Eclipse XDB-C8 (12.5 mm×2.1 mm, 5 μm), and PLRP-S (12.5 mm×2.1 mm, 15-20 μm), were tested to determine their suitability to trap DA from seawater samples. The 5 TC-C18 (2) column offered the best retention ability and good peak shape of DA, and was selected as the on-line SPE column. Validation was then performed to assess the sensitivity, linearity, matrix effects (MEs), recoveries, and precisions of the proposed method. After simple treatment of the seawater samples by filtration and acidification, 0.6 mL of the seawater sample was injected directly for on-line SPE-LC-MS/MS. The linearity was good, and ranged from 10.0 to 500.0 ng/L (correlation coefficient *R*^2^=0.9992). The limit of detection (LOD) and limit of quantification (LOQ) of DA were 4.0 and 10.0 ng/L, respectively, with good recovery (≥81.0%) and precision (RSDs≤4.2%) at three spiked levels in the blank seawater samples. After the DA in the 80.0 mL seawater sample was enriched by off-line SPE, a 0.6 mL sample was injected for on-line SPE-LC-MS/MS. The DA in the spiked blank seawater sample showed a good linear relationship in the range of 0.3-50.0 ng/L (*R*^2^=0.9990). The LOD and LOQ were 0.1 and 0.3 ng/L, respectively. The recoveries of DA at low, medium, and high spiked levels in the blank seawater samples were all ≥69.2%, and the RSDs were ≤4.4%. The MEs of DA with both methods were 18.3% and 13.7%, respectively, indicating that the ME was mild enough to be negligible. In summary, the proposed method is simple, sensitive, robust, and powerful for the detection of DA in inshore and offshore seawater.

近几十年来,随着人类活动和全球气候变化的加剧,近海赤潮灾害发生频率呈逐年上升趋势^[1]^。目前已知全球约300多种海洋赤潮藻中,至少有100种是产毒藻,而有毒赤潮暴发后会释放大量藻毒素,对海洋渔业生物造成严重危害,并且藻毒素易于通过食物链传递,进而威胁人类健康^[2,3]^。软骨藻酸(domoic acid, DA)是一种具有神经毒性的典型海洋藻毒素,具有较强的亲水性,是记忆丧失性贝毒的主要成分,主要由海洋硅藻拟菱形藻产生^[4]^。海洋环境中的DA不仅会毒害各类渔业生物,还会导致大型海洋哺乳动物及人类中毒,甚至死亡^[5,6,7,8]^,例如DA曾引起美国大量海狮死亡^[9]^,加拿大爱德华王子岛贝类中毒事件,最终导致4人死亡^[4]^。此外,当海水中DA浓度达到3.2 pmol/L时,斑马鱼胚胎心脏畸形率增加;当海水中DA浓度达到32.0 pmol/L时,斑马鱼胚胎死亡率增加并且会改变心脏基因的表达^[10]^。

对我国近海海域的伪装拟菱形藻(福氏拟菱形藻、尖细拟菱形藻、伪柔弱拟菱形藻和伪善拟菱形藻)进行分离纯化,均检测出了DA^[11,12,13]^,由此可以看出,在我国海域中,能产生DA的有毒藻较多。近海海域又是海产养殖区密集分布地,海水中的DA将威胁海产养殖业的健康发展。因此针对近海养殖区海水中的DA进行有效监测尤为重要。

目前,用于海产品中DA检测的常用方法主要包括:高效液相色谱法^[14]^、液相色谱-串联质谱法(LC-MS/MS)^[15]^、酶联免疫分析法等^[16]^。其中,LC-MS/MS具有特异性强、准确度和灵敏度高等优点,是目前DA测定最常用的方法。近海海水中DA含量通常在ng/L水平^[17,18]^,且高盐海水基质样品无法直接进行LC-MS/MS检测,因此需要对海水样品中的DA进行富集和净化。被动固相吸附法和固相萃取法(包括小柱和膜盘)是目前海水中DA富集的常用方法^[3]^,被动固相吸附法已用于美国加利福尼亚沿岸海水中DA的富集检测^[19]^,但该方法不能确定对海水中DA的富集倍数,致使无法对海水中DA进行准确定量。Wang等^[20]^采用反相C18固相萃取柱实现了20.0 mL海水中DA的富集,但通过增加海水上样量提高DA富集倍数时,DA回收率会明显下降。王九明等^[18]^利用固相萃取膜盘实现了海水中痕量DA的高效富集,富集倍数高达2000倍,然而该方法需要消耗大量海水样品(2.0 L),并且整个处理过程有机溶剂消耗量大,操作过程复杂。

近十年来,在线固相萃取技术在水环境有机污染物快速检测领域应用越来越广,实现了微囊藻毒素^[21]^、除草剂^[22]^、多环芳烃^[23]^以及脂溶性海洋藻毒素^[24]^等有机污染物的快速检测。该技术具有所需水样少、有机溶剂用量少、自动化程度高、快速高效等优点,在水环境有机污染物日常检测和监测工作中具有广阔的应用前景^[25,26]^。迄今为止,采用在线固相萃取结合液相色谱-质谱联用技术快速富集检测海水中DA的研究鲜有报道,本文通过两种前处理模式,建立了检测海水中DA的分析方法,海水样品经酸化处理后,直接进行在线SPE-LC-MS/MS测定,适用于DA含量相对较高的海水样品测定;针对DA浓度较低的海水样品,先采用离线SPE富集、净化,然后结合在线SPE-LC-MS/MS对样品进行进一步处理和分析,以实现海水中超痕量DA的测定。

## 1 实验部分

### 1.1 仪器、试剂与材料

1290 II超高效液相色谱仪(配有四元泵、二元泵、自动进样器(带大体积进样组件,最大进样量为900 μL)、柱温箱(带两位六通阀))、6470A三重四极杆质谱仪(配有喷射流电喷雾电离(ESI)源)、5 TC-C18(2)保护柱(12.5 mm×4.6 mm, 5 μm)和5 TC-C18(2)分析柱(150 mm×4.6 mm, 5 μm)(美国Agilent公司); Fotector Plus全自动固相萃取仪(睿科集团(厦门)股份有限公司); FA1104电子天平(上海精天电子仪器厂); Milli-Q超纯水处理系统(美国Millipore公司); RE100-Pro旋转蒸发仪(北京大龙公司); HLB固相萃取柱(200 mg/6 mL,纳谱分析技术(苏州)有限公司); 0.22 μm混合纤维微孔滤膜(上海新亚净化器件厂)。

色谱纯甲酸和优级纯乙酸铵(瑞士Fluka公司);色谱纯甲醇和乙腈(美国Tedia公司); DA标准品(美国Sigma公司);实验用水为自制Milli-Q超纯水(18.2 MΩ·cm)。海水样品取自青岛近海。

### 1.2 标准溶液的配制

取1.0 mg DA标准品,用5%乙腈水溶液溶解,并定容至10.0 mL容量瓶中,得到质量浓度为100.0 mg/L的DA标准储备液,于-80 ℃避光保存。用5%乙腈水溶液对DA标准储备液逐级稀释,得到质量浓度为1.0 ng/L~1.0 mg/L的系列标准溶液,于-20 ℃避光保存。

### 1.3 实验条件

1.3.1 离线固相萃取条件

移取经0.22 μm滤膜过滤的80.0 mL海水样品,加入0.32 mL甲酸进行酸化,以1.0 mL/min的流速通过HLB固相萃取柱(已用5.0 mL甲醇和超纯水活化,加载流速为1.0 mL/min),并使用5.0 mL超纯水进行淋洗,淋洗后利用氮气以160 mL/min的流速气推持续5 min;再采用10.0 mL甲醇洗脱DA,洗脱流速为1.0 mL/min;将甲醇洗脱液于50 ℃条件下旋蒸至近干,用0.8 mL 5%乙腈水溶液(含0.1%甲酸)复溶,并用0.22 μm滤膜过滤,待测。

1.3.2 在线固相萃取条件

海水样品经酸化(加入海水体积0.1%的甲酸)或经离线固相萃取后,均可直接进行在线SPE-LC-MS/MS分析。使用5 TC-C18(2)保护柱(12.5 mm×4.6 mm, 5 μm)作为在线SPE柱进行DA在线富集,进样体积为0.6 mL,样品加载流动相为5%乙腈水溶液(含0.1%甲酸),流速为1.0 mL/min。在线固相萃取六通阀切换示意图如图1所示,0~8 min时,六通阀为1-6位相通(见图1a); 8~20 min时六通阀切换至1-2位相通(见图1b); 20 min后六通阀切回1-6位相通。

**图1:**
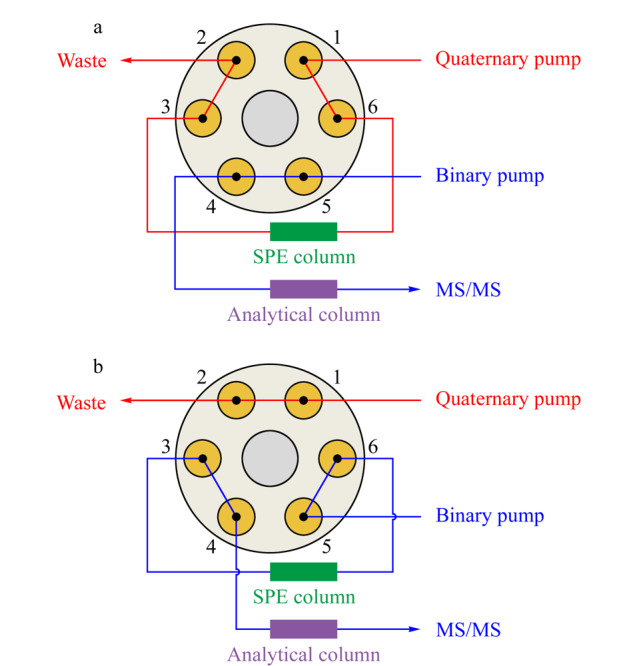
在线固相萃取时六通阀切换示意图

1.3.3 液相色谱-串联质谱条件

色谱柱5 TC-C18 (2)分析柱(150 mm×4.6 mm, 5.0 μm),柱温25 ℃,流动相(A)水和(B)90%乙腈水溶液(均含2.0 mmol/L乙酸铵和0.2%甲酸),流速0.5 mL/min。梯度洗脱程序:0~8 min, 3%B; 8~18 min, 3%B~100%B; 18~20 min, 100%B。

离子源:ESI源、正离子模式,毛细管电压:4000 kV;鞘气流速:11 L/min;鞘气温度:340 ℃;干燥气流速:7 L/min;干燥气温度:300 ℃;雾化器压力:310.6 kPa;多反应监测(MRM)模式;DA母离子*m/z*为312.1,碎裂电压130 V;子离子*m/z*分别为248.2(定性)和266.2(定量),碰撞能量分别为18 eV和16 eV。

## 2 结果与讨论

### 2.1 色谱条件的优化

根据前期^[18]^研究结果,5 TC-C18(2)色谱柱对DA有良好的分离效果,因此本文采用5 TC-C18(2)分析柱用于DA的色谱分离。

甲醇水溶液^[27]^和乙腈水溶液^[28]^常被用作DA液相色谱分离的有机流动相,于是对90%甲醇水溶液(含2.0 mmol/L乙酸铵和0.2%甲酸)和90%乙腈水溶液(含2.0 mmol/L乙酸铵和0.2%甲酸)作为有机流动相时DA的LC-MS/MS分析结果进行了比较。如图2a所示,90%甲醇水溶液对DA有更强的洗脱能力,但目标物响应相对较低;90%乙腈水溶液作为有机流动相时,DA响应更高,具有更好的仪器检测灵敏度。由于DA在质谱中易生成[M+H]^+^母离子,流动相中酸性程度影响化合物离子化效率,进而影响方法的灵敏度。本文对流动相中添加不同体积分数甲酸(0.1%、0.2%、0.3%和0.4%)的效果进行了比较(见图2b),可以看出,甲酸体积分数为0.2%时,DA的响应最高。因此选择90%乙腈水溶液(含2.0 mmol/L乙酸铵和0.2%甲酸)为有机流动相。

**图2:**
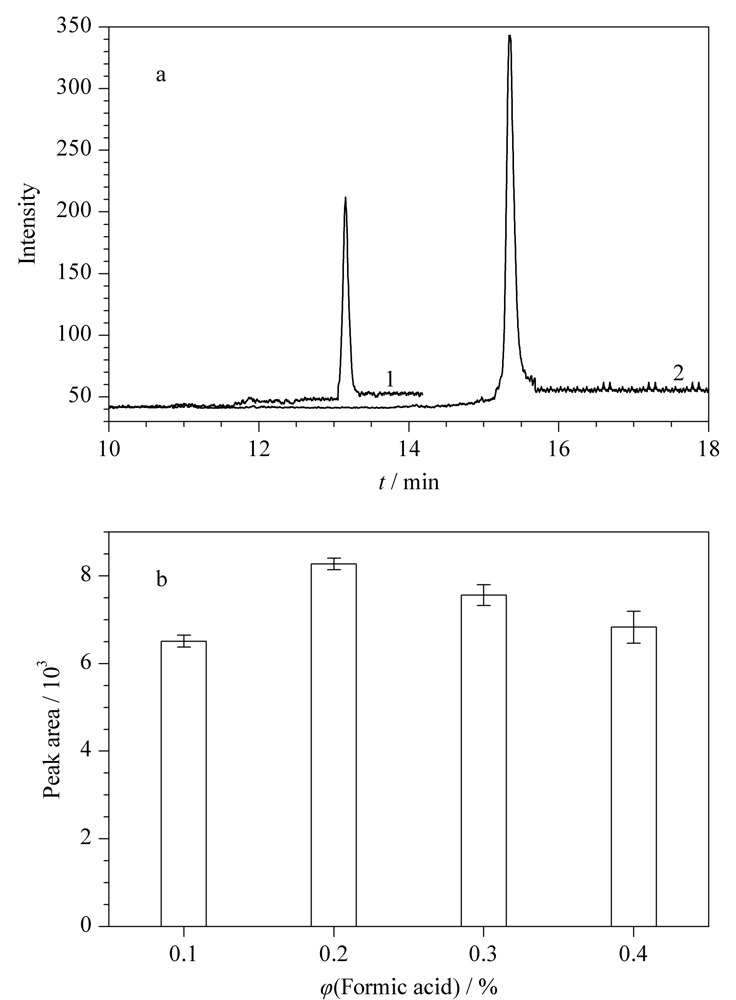
(a)不同有机流动相体系时DA的色谱图和(b)流动相中添加不同体积分数甲酸时DA的峰面积(*n*=3)

### 2.2 质谱条件的优化

采用ESI源分析DA,在正离子模式条件下DA易生成*m/z* 312.2 [M+H]^+^特征离子峰,负离子模式下易生成*m/z* 310.2 [M-H]^-^特征离子峰。但正离子模式条件下DA的检测灵敏度更高,因此选择在正离子模式下进行海水样品中DA的分析。

随后,在MRM模式下,对影响母离子(*m/z* 312.2)和子离子丰度的两个主要参数破碎电压和碰撞能量进行了优化。破碎电压为130 V时,方法灵敏度最高;通过调节碰撞能量,选取DA二级质谱分析的最优子离子,以信号最强的子离子(*m/z* 266.2)为定量离子,信号次强的子离子为定性离子(*m/z* 248.2),并通过优化各离子对的碰撞能量,最终确定最优碰撞能量为16 eV(*m/z* 266.2)和18 eV *m/z* 248.2)。

### 2.3 离线固相萃取条件的优化

在离线固相萃取时,选择能高效富集DA的固相萃取柱至关重要。HLB固相萃取柱填料为聚乙烯吡咯烷酮聚合物,对极性和非极性化合物均有较好的富集效果,因此选择HLB固相萃取柱用于海水中DA的离线富集净化。因DA属于弱酸性化合物,在海水中易发生电离,不易于HLB固相萃取柱对其吸附,因此,可通过酸化海水样品抑制其电离,提高HLB小柱对DA的富集效率。海水加标样品(10.0 ng/L)添加不同体积的甲酸,海水酸化处理后DA的回收率明显提高,当甲酸的体积为0.32 mL时,HLB固相萃取柱对DA的吸附效率最高(见图3)。这与王九明等^[19]^采用磺化苯乙烯-乙烯基苯共聚物(SDB-RPS)固相萃取膜盘富集海水中的DA所添加的甲酸体积相一致。

**图3:**
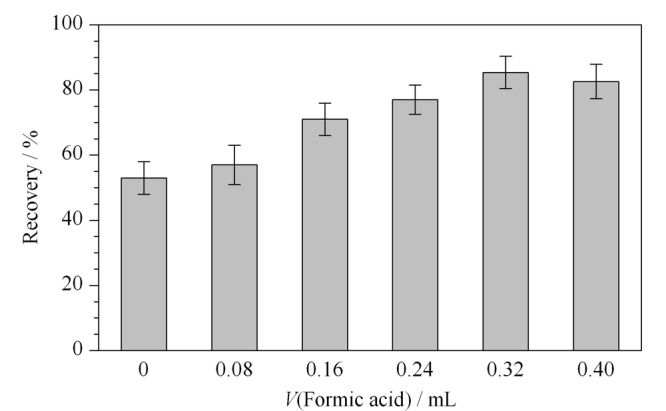
不同甲酸体积对海水中DA回收率的影响(*n*=3)

### 2.4 在线固相萃取条件的优化

采用在线固相萃取用于水样中有机化合物的富集时,首先是选择填料类型合适、富集净化效果良好的富集柱。据相关文献^[20]^可知,反相C18填料的固相萃取柱可以富集海水中的DA,基于此,实验对4种不同填料的在线固相萃取柱进行了比较(见图4), 4种富集柱分别为美国Agilent公司的5 TC-C18(2)(12.5 mm×4.6 mm, 5 μm)、Zorbax Eclipse Plus-C18(12.5 mm×2.1 mm, 5 μm)、Zorbax Eclipse XDB-C8(12.5 mm×2.1 mm, 5 μm)和PLRP-S(12.5 mm×2.1 mm, 15~20 μm)柱。

**图4:**
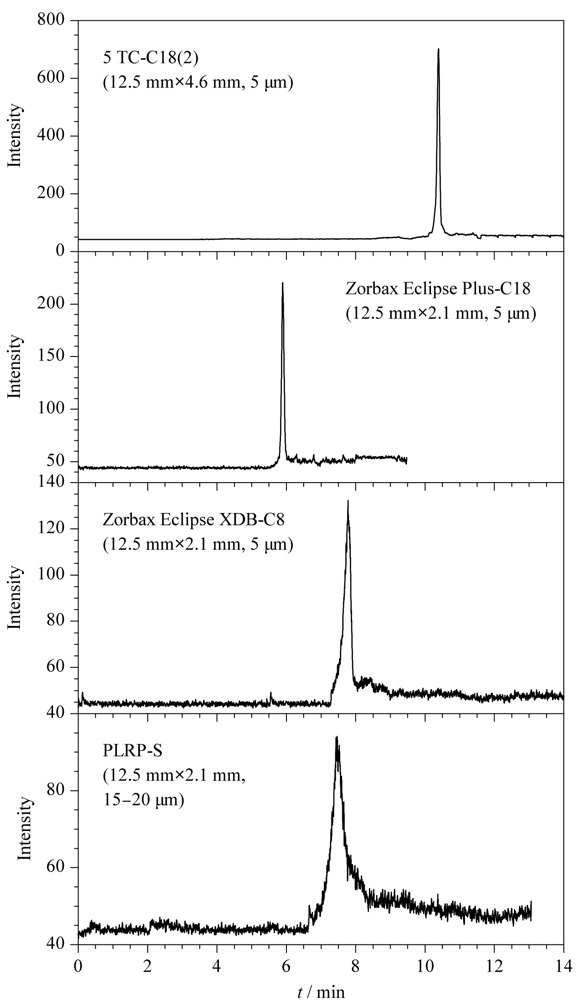
不同在线固相萃取柱对DA保留能力的影响

5 TC-C18(2)柱对DA有更好的保留能力,峰形较好并且质谱响应较高;DA在Zorbax Eclipse Plus-C18在线固相萃取柱上峰宽较窄,但质谱响应较低;DA在Zorbax Eclipse XDB-C8和PLRP-S在线固相萃取柱上会产生扩散现象,导致峰宽较宽。因此,实验选择5 TC-C18(2)柱作为测定DA的在线固相萃取柱。

实验还考察了海水样品酸化处理对5 TC-C18(2)柱在线富集DA的影响,加入相对海水体积0.1%的甲酸时,DA的富集率最高、色谱峰面积最大,因此最终确定了加入甲酸的体积为海水体积的0.1%。

### 2.5 方法学考察

本文对两种检测模式下方法的基质效应(ME)、线性范围、灵敏度和回收率等进行了考察。

2.5.1 基质效应评价

ME在LC-MS/MS分析中普遍存在,主要表现为对目标化合物的检测信号有增强或者抑制作用,从而影响目标化合物的定量准确度。ME=|基质溶液中DA的峰面积-纯水溶液中DA的峰面积|/纯水溶液中DA的峰面积。ME可分为3个等级:(a)0~20%,轻微影响;(b)20%~50%,中度影响;(c)大于50%,显著影响^[29]^。

本文分别采用超纯水和空白海水配制3个水平(20.0、80.0和300.0 ng/L)的DA加标样品,每个水平制备3个平行样品,通过在线SPE-LC-MS/MS和离线SPE结合在线SPE-LC-MS/MS测定比较,考察ME对检测结果的影响。在这两种检测模式下,平均ME分别为18.3%(低、中和高水平基质效应:19.7%、18.5%和16.7%)和13.7%(低、中和高水平基质效应:15.1%、14.2%和11.8%), DA质谱信号均受到轻微影响,因此在采用在线SPE-LC-MS/MS和离线SPE结合在线SPE-LC-MS/MS测定海水DA过程中可以忽略ME的影响。

2.5.2 线性方程

将DA标准储备液逐级稀释,得到质量浓度分别为0.3、1.0、3.0、10.0、20.0、50.0、100.0、200.0和500.0 ng/L的系列标准溶液,以DA的质量浓度(ng/L)为横坐标(*x*, ng/L)、定量离子峰面积为纵坐标(*y*)绘制标准曲线。在10.0~500.0 ng/L范围内,直接在线SPE-LC-MS/MS检测模式下的线性方程为*y*=7.4311*x*+188.68(线性相关系数*R*^2^=0.9992);在0.3~50.0 ng/L范围内,离线SPE结合在线SPE-LC-MS/MS检测模式下的线性方程为*y*=524.07*x*+163.87(*R*^2^=0.9990)。结果表明这两种检测模式下线性范围内线性关系良好。

2.5.3 检出限和定量限

在空白海水样品中加入低水平的DA标准溶液,分别采用两种检测模式考察方法的灵敏度,将DA信噪比(*S/N*)为3和10时所对应的质量浓度作为方法的检出限(LOD)和定量限(LOQ)。海水样品直接采用在线SPE-LC-MS/MS测定,LOD和LOQ分别为4.0 ng/L和10.0 ng/L,已达到或优于表1所列文献中方法的灵敏度,能满足近海海水中DA的常规监测需求。离线SPE结合在线SPE-LC-MS/MS测定,方法LOD和LOQ分别为0.1 ng/L和0.3 ng/L,明显优于其他文献^[18,30,31]^,可用于远岸海水中超痕量DA的测定以及开展海水中DA降解规律等对检测灵敏度要求较高的研究工作。

**表1:** 本方法与文献方法的比较

Pretreatment technique	LOD/(ng/L)	LOQ/(ng/L)	Recovery/%	RSD/%	Sample volume/(mL)	Ref.
SDB-RPS SPE disk	2.5	10.0	≥89.3	≤4.0	2000.0	[18]
C18 SPE disk	20.0	60	≥101.4	≤8.4	20.0	[30]
C18 SPE column	5.0	10.0	≥90.0	≤8.0	50.0	[31]
Direct on-line SPE	4.0	10.0	≥81.0	≤4.2	0.6	this work
Off-line SPE-on-line SPE	0.1	0.3	≥69.2	≤4.4	80.0	this work

2.5.4 回收率和精密度

取空白海水样品,采用标准加入法对方法的回收率和精密度进行考察。结果表明,直接采用在线SPE-LC-MS/MS测定低、中、高水平(20.0、100.0和300.0 ng/L)DA海水加标样品,每个加标水平制备6个平行样(*n*=6), DA的平均加标回收率分别为81.0%、83.5%和86.6%, RSD分别为4.2%、4.1%和3.8%,表明在该检测模式下方法回收率和精密度均良好。离线SPE结合在线SPE-LC-MS/MS测定模式下,方法的加标回收率在低、中、高(1.0、5.0和20.0 ng/L)水平下分别为69.2%、71.7%和78.5%, RSD分别为4.4%、4.3%和3.1%,说明两步固相萃取可满足海水中的DA有效富集,并且方法的精密度良好,满足实际海水中DA测定的准确度要求。

## 3 结论

本文建立了在线SPE-LC-MS/MS和离线SPE结合在线SPE-LC-MS/MS测定海水中DA的分析方法。海水样品经酸化处理后可直接进样分析,该方法操作简单,自动化程度高,节省溶剂,较小体积海水样品即可满足近海海水中DA常规监测的灵敏度要求。海水样品经离线SPE结合在线SPE-LC-MS/MS大体积进样分析,具有更低的灵敏度,可为大洋或极地海水中超痕量的DA测定提供可靠的技术支撑。另外,所发展的技术方法还可推进海水中DA检测方法相关标准的建立,为海水环境中限量标准的制定和毒理研究提供依据。
